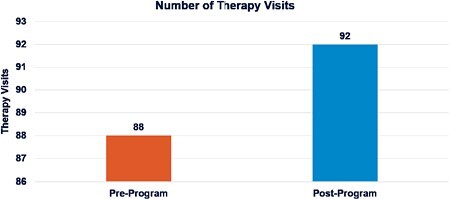# 593 Pilot Study: Self-scheduling Therapy Increases Burn Patients' Activity Tolerance and Compliance

**DOI:** 10.1093/jbcr/irae036.227

**Published:** 2024-04-17

**Authors:** Kara L Bell

**Affiliations:** HCA Tristar Skyline Medical Center, Goodlettsville, TN

## Abstract

**Introduction:**

During a hospital visit, patients often lack autonomy in making decisions for aspects of their patient care (e.g., meal times, medication management, procedures, dressing changes, labs/vitals, etc.). Prior research shows that patient autonomy in clinical decision making can yield positive results in treatment related-decisions (Jonsen et al., 2002). The purpose of this pilot study was to study the effect of having patients schedule their own therapy timing on PT/OT activity compliance and tolerance in a burn population.

**Methods:**

This is a prospective pilot study conducted on weekdays from January through March of 2023. Study participants consisted of all adult burn admissions with TBSA >5%. Patients with necrotizing fasciitis, Stevens Johnson syndrome, and toxic epidermal necrolysis with therapy consults were also included. Patients were excluded if intubated and re-assessed following extubation once alert and oriented. All aspects of the study were completed by the Burn Coordinator on staff. As part of this pilot project, each patient was evaluated by PT/OT and given the opportunity to choose a therapy time for all following visits as appropriate. Therapy schedules were subject to be adjusted based on medical holds and restrictions. Once the therapy time was determined, a laminated sheet was placed on each patient’s door identifying the therapy time for the day to coordinate with other disciplines. Next, a therapy log was compiled and audited weekly by the Burn Coordinator. Nursing staff were also educated at shift change by the Burn Coordinator. The Burn Coordinator maintained a printed schedule with patient times and had the ability to adjust scheduling due to time conflicts, admissions, and discharges. Patients not requiring a co-treatment were seen sequentially by PT/OT to maintain consistency with a self-scheduled timeline. Participant’s PT/OT compliance and tolerance post-pilot will be compared to their compliance and tolerance pre-pilot (October-November 2022) where therapists were responsible for patient scheduling per their availability.

**Results:**

Participant’s PT/OT compliance and tolerance was compared to the prior standard of care at the institution. Prior to the pilot study, there was a higher incidence of declining therapy services for PT/OT when the provider chose the time. During study there was a decrease in decline of therapy services, increased time tolerated during session, increased total number of visits, and increase in early mobility. The number of therapy visits pre vs post study was found to be statistically significant.

**Conclusions:**

Patient autonomy in therapy selection time leads to increased tolerance and compliance in a burn patient population.

**Applicability of Research to Practice:**

-Identifies the need for self scheduling to increase functional outcomes

-The project empowered the burn patients to have autonomy within their acute care stay